# Effect of extracurricular tutoring on adolescent students cognitive ability: A propensity score matching analysis

**DOI:** 10.1097/MD.0000000000035090

**Published:** 2023-09-08

**Authors:** Qi Zhang, Jiafei Yang, Wenlong Wang, Zhihong Liu

**Affiliations:** a School of Public Health, Ningxia Medical University, Yinchuan, China; b Department of Epidemiology and Health Statistics, School of Public Health, Ningxia Medical University, Yinchuan, China; c Department of Occupational and Environmental Health, School of Public Health, Ningxia Medical University, Yinchuan, China.

**Keywords:** adolescent students, cognition, extracurricular tutoring, propensity score matching

## Abstract

In recent years, there has been a substantial increase in the number of adolescent students attending extracurricular tutoring. However, extracurricular tutoring, being a distinct form of education, may have varying effects on cognitive capabilities compared to conventional education. Accordingly, the purpose of this paper is to conduct a thorough examination of the effects of extracurricular tutoring on cognitive abilities among adolescent students. The study utilized national data from the China Family Panel Study 2018 to explore the relationship between involvement in extracurricular tutoring among students and cognitive abilities. The study included 2567 valid respondents. A binary logistic regression model was built to determine the factors associated with tutoring involvement while controlling for 19 individual, family, and school factors. Furthermore, a propensity score-matched analysis was conducted in order to mitigate potential bias by using confounding variables identified in the previous step. The study results show that participation in extracurricular tutoring can significantly increase the level of cognitive ability of adolescent students, with adjustments made for age, gender, ethnicity, number of family members, net family income per capita, education and training expenditure in the past years, change of residence for enrollment, change of domicile address for enrollment, locality of the current school, class size, hold a position as a class cadre, average daily study time on weekdays, average study time per day during weekends. The findings imply that the government should provide tutorial subsidies to disadvantaged groups of adolescent students, allocate educational resources equitably, and invest more in education resources in less developed regions to foster fair and healthy development of education and improve the cognitive abilities of young students in the long-term.

## 1. Introduction

Participating in extracurricular tutoring has emerged as a global educational phenomenon, particularly in China.^[1,2]^ This practice is shaped by various socio-cultural factors beyond the education system, including social competition and family expectations, cultural attitudes towards education, and traditional beliefs about knowledge and learning. In particular, in many East and South Asian countries, socio-cultural expectations and norms foster intense competition and high levels of stress among parents and students that drive the demand for extracurricular tutoring to enhance academic performance and skill acquisition. Besides, in some cultural contexts, better access to knowledge and educational opportunities is considered critical for personal success; as a result, extracurricular tutoring is viewed as a mechanism for improving learning outcomes and boosting competitive advantages. In certain Middle Eastern and Asian nations, extracurricular tutoring is viewed positively, with strong cultural values placing a high premium on education and learning. The multifaceted cultural impact of extracurricular tutoring reflects a combination of factors, including social, familial, and educational dimensions, and its effects on the long-term development of education and society remain uncertain. Despite the issuance of several government regulations in recent years to limit extracurricular tutoring, it continues to be a pervasive and popular phenomenon due to the growing competition for better life opportunities and the perception of the inadequacy of traditional school education to meet these challenges.^[3]^

Extracurricular tutoring refers to diverse educational activities that take place outside regular school hours, including subjects such as language, mathematics, English, music, sports, and art lessons, among others.^[4,5]^ Various types of extracurricular tutoring are emerging, ranging from personalized 1-on-1 sessions to group classes, online courses, homework clubs, and camp-style programs.^[6,7]^ While some studies have shown that the average effect of private tutoring is not significant, it may have notable and positive effects on specific subjects for urban students with lower academic achievement or those in low performing schools.^[8]^ Recently, the government has been emphasizing the need to appropriately reduce the academic burden on primary and secondary school students, and has stressed the importance of schools cultivating students interests and innovative spirit comprehensively. The government has also issued measures to enforce these requirements in schools. Schools have responded accordingly by implementing reforms, such as reducing the amount of in school class time and adding courses that help develop students interests. However, due to the competitive educational environment, extracurricular tutoring is still very popular in primary and secondary schools.^[9]^ While research has emphasized the significance of cognitive development in educational outcomes, there is limited knowledge of the effects of extracurricular tutoring on this development, as it varies from traditional teaching methods. Therefore, it is essential to undertake more research on extracurricular tutoring to gain a better understanding of its impact on students cognitive development.

extracurricular tutoring plays a crucial role in enhancing students’ cognitive capabilities. Cognitive ability encompasses a range of skills, including the ability to process, store, and extract information. It provides people with the capacity to comprehend the composition of things, the relationship between performance and other stimuli, the driving force, the direction of development, and fundamental laws.^[10–12]^ Furthermore, cognitive ability constitutes the foundation for completing activities^[13]^ and can be divided into general and specific cognitive ability.^[14]^ The former is generally referred to as intelligence, while the latter includes language, memory, calculation, reasoning, decision-making, and spatial abilities.^[15]^ Therefore, cognitive ability is fundamental to learning, and it partly determines an individual’s developmental prospects.^[16]^ The impact of extracurricular tutoring on cognitive development is often manifested through the provision of additional learning opportunities, building a broad knowledge network, strengthening the analytical and problem-solving skills, improving memory, and cultivating good study habits.

Although extracurricular tutoring has been associated with positive outcomes on cognitive development, the impact of such programs on adolescent students is complex and influenced by several factors. There is limited research in this area, which can be attributed to various reasons. Firstly, there is a lack of a unified evaluation system and tools for research and comparison, as extracurricular tutoring comes in various forms.^[17]^ Secondly, there is sample selection bias, as clients come from different family backgrounds and levels of education and economic status, making it challenging to compare different young people randomly. Finally, there is diversity in extracurricular tutoring, including the timing, form, and content of tutoring, which limits the ability to conduct specific breakdowns and analysis.^[17]^ This complexity has resulted in the need for methods such as propensity score matching (PSM) to control for confounding variables that may influence the results of research studies.

In this study, to overcome these problems, we have used the cognitive ability test results from the China family panel studies (CFPS),^[18,19]^ and PSM to investigate the impact of extracurricular tutoring on young people’s cognitive abilities. The PSM method is a statistical technique that controls for jointly dependent covariates that affect decisions to procure extracurricular tutoring and cognitive development. In doing so, PSM reduces the selection bias caused by confounding variables across the 2 different groups of young people. PSM has become an emerging technique for causal inference in observational research and is increasingly being used to eliminate the imbalance between intervention and nonintervention groups, as well as reduce selection bias caused by confounding variables.^[20–26]^ The selection of appropriate confounding variables is, however, crucial in the application of PSM. In our study, the confounding variables selected aligned with teenagers’ cognitive ability, and their involvement in extracurricular tutoring was included in the study.

The literature suggests that engaging in extracurricular tutoring is influenced by individual, family, and school factors.^[27]^ Individually, students with higher grades, those living with their parents, and day students tend to be more likely to engage in extracurricular tutorials.^[28,29]^ From a family perspective, factors that include parental education and family income play a crucial role in whether students attend extracurricular tutoring or not.^[7,30]^ In terms of school characteristics, students who attend central city schools and public schools with higher rankings tend to participate in extracurricular English tutorial classes.^[28]^ Furthermore, the proportion of urban students who take extracurricular tuition is significantly higher than that of rural students, with a difference of 26.31%.^[31]^

After reviewing the literature, we noted that confounding variables can adversely affect the relationship between adolescent participation in tutoring and cognitive ability. These variables contribute to observed data selection bias, making it difficult to conclude whether participation in extracurricular tutoring affects adolescents’ cognitive abilities. However, adopting the propensity score matching method can improve the methodological standards and help researchers make stronger causal inferences from observational data.^[32]^ Although PSM method has its advantages, it is not the ultimate solution to all endogeneity problems. However, if applied appropriately, PSM can effectively reduce selection bias between the treatment and control groups. The PSM method involves matching adolescents not involved in extracurricular tutoring with the control group, balancing the treatment group. By calculating the average treatment effect on the treated (ATT) between the treatment and control groups for those treated, researchers can provide stronger evidence to validate the effect of extracurricular tutoring on adolescents cognitive abilities. In this study, we identified 19 relevant confounding variables and selected them synthetically as covariates for PSM based on relevant literature and variable availability.

## 2. Methods

### 2.1. Sampling

This study analyzed the data from the CFPS (https://opendata.pku.edu.cn/dataverse/CFPS), a social survey sponsored by the Institute of Social Science Survey of Peking University. Launched in 2010, the CFPS permanently tracked all family members and their future biological or adopted children^[33–35]^ to obtain reliable data for academic research. The survey covered 25 provinces, municipalities, and autonomous regions, with a target sample size of 16,000 households. This study focused on young people aged between 9 and 18 years old, using the 2018 Individual, Family, and Household Economy database. The family information in the adult pool was first matched against the parent sample number in the individual pool, and then against the family sample number of the individual’s family to obtain the family economic information.

In order to achieve the research objectives of this paper, sample cases were selected based on data from the sample of young people and parents in the CFPS survey using the following criteria: Exclusion of cases with missing values for selected variables; Exclusion of individual cases with learning disabilities, intellectual disabilities or other cognitive impairments; Exclusion of cases where answers did not match reality, such as those with sleep hours exceeding 12 or <4 hours, those with an average of more than 6 hours of extracurricular tutoring per day from Monday to Friday, and cases with an average of more than 12 hours of extracurricular tutoring per day on weekends. The final valid sample size obtained was 2567. The CFPS was reviewed and approved by the Biomedical Ethics Committee of Peking University, and the data collection was carried out with the approval of the ethical review. The approval number is IRBO000105214010.

## 3. Measurement

### 3.1. Cognitive function

The cognitive measures used in this study were based on a separately designed cognitive module in the CFPS questionnaire. The module used a set of word test and a set of math test to examine the cognitive level of the respondents. The word test assessed the linguistic level of the respondents by flipping the tab to the appropriate position and asking respondents to pronounce the word or phrase in that position. The math test included 4 different sets of mathematical questions, with 1 set randomly selected for respondents to answer. This measure of cognitive level provided a more comprehensive picture of the respondent’s cognitive ability.

Cognitive ability was quantified by the respondents’ word test scores (ranging from 0–34) and math test scores (ranging from 0–24).^[19,36]^ A higher score indicated a better level of cognitive development.

### 3.2. Extracurricular tutoring

extracurricular tutoring is a paid educational service provided by private individuals or training institutions to schoolchildren outside of regular school hours with the aim of improving academic performance or specific skills, such as art, sports, or academic competition. extracurricular tutorials in this study included both curriculum tutorials aimed at improving academic performance and comprehensive quality development aimed at developing students’ specific skills in a particular area. The 2018 CFPS database included a question regarding participation in tutoring or tutoring classes. Respondents who answered “yes” were considered to have participated in extracurricular tutoring. Of the 2567 eligible participants, 564 had participated in extracurricular tutoring.

### 3.3. Covariates

For this study, 19 questions from the CFPS database were selected as covariates that might affect the cognitive development and decision-making of adolescent students in relation to extracurricular tutoring. The questions covered 3 main aspects: including: adolescents characteristics, such as gender, age, and ethnicity; family characteristics like the number of family members and family financial situation; and school characteristics such as whether the adolescent attends a public school or not and currently enrolled in which stage of education. Table 1 displays the 19 confounding covariates used in this study and their attributes.

**Table 1:** Covariate measurement and attributes.

Covariates	Measurements	Type	Abbreviation
Age (yr)	Range from 9 to 18	A continuous variable	Age
Gender	1 = male vs 0 = female	A dichotomous variable	Gender
Ethnicity	1 = Han ethnicity vs 0 = other ethnicities	A dichotomous variable	Ethnicity
Number of family members	Range from 1 to 15	A continuous variable	NOFM
Net family income per capita (KCNY)	Range from 0 to 540	A continuous variable	NFIPC
Education and training expenditure in the past year (KCNY)	Range from 0 to 140	A continuous variable	EATEITPY
Change of residence for enrollment	1 = yes vs 0 = no	A dichotomous variable	CORFE
Change of domicile address for enrollment	1 = yes vs 0 = no	A dichotomous variable	CODAFE
Currently enrolled in school	1 = yes vs 0 = no	A dichotomous variable	CEIS
Currently enrolled in which stage of education	1 = middle school, 0 = primary school	A dichotomous variable	CEIWSOE
Locality of the current school	0 = rural, 1 = urban	A dichotomous variable	LOTCS
Public school	1 = yes vs 0 = no	A dichotomous variable	PS
Enroll in the nearest school	1 = yes vs 0 = no	A dichotomous variable	EITNS
Class size	Range from 2 to 119	A continuous variable	CS
Hold a position as a class cadre	1 = yes vs 0 = no	A dichotomous variable	HAPAACC
Average daily study time on weekdays (h)	Range from 0 to 24	A continuous variable	ADSTOW
Average study time per day during weekends (h)	Range from 0 to 24	A continuous variable	ASTPDDW
Have you ever requested leave from school	1 = yes vs 0 = no	A dichotomous variable	HYERLFS
Have you ever skipped class	1 = yes vs 0 = no	A dichotomous variable	HYESC

## 4. Statistical analysis

Data analysis was performed using Stata 15.1 SE. Normally distributed continuous variables were expressed as means ± standard deviations, and categorical variables were expressed as frequencies or percentages. Two independent samples *t* tests were used for continuous variables, and the Chi-square test was used for categorical variables.^[37]^ Missing continuous variables were imputed using means or medians.^[38]^

To address selective bias in the observed data, PSM was used to establish cohorts with similar baseline characteristics. PSM is a statistical technique that measures the effects of an intervention without the need to randomize the subjects of the intervention. Augmenting the foundational information, the paper elaborates on the PSM method by explaining the necessary steps to conduct the analysis. The first step is to calculate a propensity score, and the second step is to match against the propensity score to estimate the impact of the ATT.^[39,40]^ Thus, statistical analyses were conducted using Psmatch2 in Stata 15.1 SE to assess the relationship between participation in extracurricular tutoring and adolescent cognitive abilities. To start, we estimated a binary logistic regression model using participation in extracurricular tutoring as the dependent variable and 19 individual, school, and family factors as independent variables. Next, we conducted propensity score analysis to control potential bias using confounding covariates identified in the previous step. For this analysis, PSM used concepts and terminology from experiments, considering the nontreatment unit as the control group and the treatment unit as the intervention group. In our study, the “control group” consisted of adolescents who did not participate in extracurricular tutoring, and the “intervention group” consisted of those who did participate.

Moreover, to compare cognitive abilities between adolescents who did and did not participate in extracurricular tutoring, paired *t* tests were conducted for the propensity score matching group. We used 3 common matching methods, kernel matching, radius matching, and nearest neighbor matching, between the steps of matching and estimating ATT, to ensure the reliability of the results.^[24,41]^ Traditional ATT estimation produces erroneous standard errors due to the high correlation between matched samples and the error between propensity values and actual values. To combat this issue, empirical standard errors were obtained by using the bootstrap method. For the treatment group, this study used the bootstrap method to estimate ATT and empirical standard errors.^[42]^

## 5. Results

### 5.1. Descriptive statistics

This study presents an overview of the participants’ basic characteristics and provides a descriptive analysis of 2 distinct sample groups. Meanwhile, we conducted a comparison of the differences between the 2 sample groups, as depicted in Table 2. After dealing with missing values and outliers, a total of 2567 participants were eligible for this study. The participants average age was 13.534 ± 2.460 years, and there were more male (1363) than female (1204) participants. Majority (87.03%) of the respondents were Han Chinese, and the average number of household members was 4.98 ± 1.76, with a net family income per capita of RMB 17.917 ± 25.081 thousand, and an education and training expense of RMB 8.761 ± 11.301 thousand in the past year. A total of 55.01% of the respondents were middle school students, and 77.95% were currently studying in rural areas. The average class size was 47.97 ± 15.68, and 32.92% of the respondents held a class cadre position. The average daily study time on weekends and weekdays was 3.80 ± 2.98 hours and 8.75 ± 2.88 hours, respectively, among the participants. Out of 2567 participants, a total of 564 took part in extracurricular tutoring. Participating in these activities showed a higher level of cognitive ability among adolescent students. However, it is not straightforward to infer a causal relationship between extracurricular activities and cognitive ability.

**Table 2:** Descriptive characteristics of the total sample and the pre-matching participated groups versus not Participated groups.

	Total sample		Participated		Not participated		Between the tutored and non-tutored groups		
	N = 2567		N = 564		N = 2003				
	Mean	SD	Mean	SD	Mean	SD	Δ Mean	t	*P* value
Word test	24.800	6.289	26.168	5.052	24.415	6.545	1.753	−5.888	<.001
Math test	12.801	5.084	13.966	4.533	12.473	5.183	1.493	−6.205	<.001
Age (yr)	13.534	2.460	13.278	2.342	13.607	2.488	0.211	2.803	.005
Number of family members	4.982	1.763	4.686	1.721	5.066	1.767	−0.38	4.535	<.001
Net family income per capita (KCNY)	17.917	25.081	28.123	35.884	15.067	20.211	13.056	−11.091	<.001
Education and training expenditure in the past year (KCNY)	8.761	11.301	12.456	14.950	7.728	9.806	4.728	−8.838	<.001
Class size	47.972	15.682	49.937	14.301	47.418	16.009	2.519	−3.375	<.001
Average daily study time on weekdays (h)	8.745	2.882	9.284	2.875	8.595	2.867	0.689	−5.052	<.001
Average study time per day during weekends (h)	3.799	2.981	4.812	3.081	3.515	2.890	1.297	−9.276	<.001
	Percent		Percent		Percent		Δ Percent	χ^2^	*P*-value
	=1	=0	=1	=0	=1	=0			
Gender	0.531	0.469	0.465	0.535	0.550	0.450	−0.085	12.808	<.001
Ethnicity	0.870	0.130	0.938	0.062	0.851	0.149	0.087	29.316	<.001
Change of residence for enrollment	0.055	0.945	0.085	0.915	0.046	0.954	0.039	12.681	<.001
Change of domicile address for enrollment	0.011	0.989	0.025	0.975	0.007	0.993	0.018	12.973	<.001
Currently enrolled in school	0.959	0.041	0.959	0.041	0.958	0.042	0.001	0.005	.945
Currently enrolled in which stage of education	0.550	0.450	0.553	0.447	0.549	0.451	0.004	0.029	.101
Locality of the current school	0.779	0.221	0.420	0.580	0.164	0.836	0.256	167.751	<.001
Public school	0.912	0.088	0.915	0.085	0.911	0.089	0.004	0.099	.753
Enroll in the nearest school	0.733	0.267	0.730	0.270	0.733	0.267	−0.003	0.019	.891
Hold a position as a class cadre	0.329	0.671	0.390	0.610	0.312	0.688	0.078	12.137	<.001
Have you ever requested leave from school	0.151	0.849	0.149	0.851	0.151	0.849	−0.002	0.012	.914
Have you ever skipped class	0.175	0.825	0.012	0.988	0.019	0.981	−0.007	1.100	.294

Continuous variables were analyzed by *t* test, and categorical variables were analyzed by chi-square test.

SD = standard deviation.

Nonetheless, there were significant differences between the 2 student groups on 13 covariates. According to the study, students who were younger, had fewer family members, had higher per capita household income and higher education and training expenditure in the last year, had larger class sizes, had more average weekly study time, were female, were Han Chinese, had changed their residential and domicile addresses for further studies, attended urban schools, and were class cadres were more involved in extracurricular tutoring and had higher cognitive ability. This finding suggests that a causal relationship cannot simply be inferred between adolescent students’ participation in extracurricular tutoring and their level of cognitive ability.

### 5.2. Propensity score

First, logistic regression was performed with participation in extracurricular tutoring as the dependent variable and 19 covariates as independent variables. All variables showed statistical significance except for: age, change of residence for enrollment, change of domicile address for enrollment, currently enrolled in school, enroll in the nearest school, have you ever requested leave from school, and have you ever skipped class. These results suggest that the model is strongly predictive of whether adolescents participate in extracurricular tutoring, as shown in Table 3. The regression results were then used to build a predictive model to calculate the propensity score of all youth participating in extracurricular tutoring in the sample. The higher the propensity score, the greater the likelihood that adolescents will engage in extracurricular tutoring.

**Table 3:** Logistic regression estimates of adolescent students participating in extracurricular tutoring (N = 2567).

Variables	OR	Std. error	z-value	*P* value	95% CI
Age (yr)	0.941	0.045	−1.27	.203	[0.856,1.034]
Gender	0.718‡	0.077	−3.09	.002	[0.582,0.886]
Ethnicity	2.021‡	0.41	3.47	.001	[1.359,3.007]
Number of family members	0.908‡	0.032	−2.77	.006	[0.849,0.972]
Net family income per capita (KCNY)	1.128‡	0.029	5.00	<.001	[1.082,1.197]
Education and training expenditure in the past year (KCNY)	1.271‡	0.063	4.82	<.001	[1.153,1.402]
Change of residence for enrollment	1.405	0.304	1.57	.115	[0.92,2.147]
Change of domicile address for enrollment	1.759	0.806	1.23	.218	[0.717,4.317]
Currently enrolled in school	0.990	0.269	−0.04	.970	[0.581,1.687]
Currently enrolled in which stage of education	0.657‡	0.106	−2.60	.009	[0.479,0.902]
Locality of the current school	2.96‡	0.362	8.87	<.001	[2.329,3.763]
Public school	1.577†	0.317	2.27	.023	[1.064,2.338]
Enroll in the nearest school	1.153	0.152	1.08	.281	[0.89,1.494]
Class size	1.009‡	0.003	2.69	.007	[1.003,1.016]
Hold a position as a class cadre	1.263†	0.139	2.12	.034	[1.018,1.567]
Average daily study time on weekdays (h)	1.046†	0.022	2.13	.033	[1.004,1.091]
Average study time per day during weekends (h)	1.112‡	0.020	5.81	<.001	[1.073,1.153]
Have you ever requested leave from school	1.043	0.156	0.28	.779	[0.778,1.398]
Have you ever skipped class	1.055	0.492	0.12	.908	[0.423,2.632]

*P < .05.

† *P* < .01.

‡ *P* < .001.

### 5.3. Matching and balanced test

**Figure 1.**
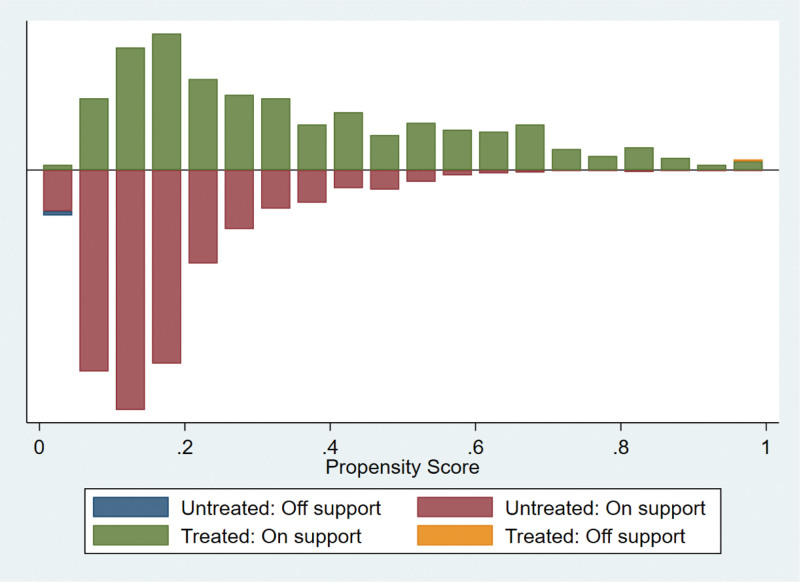
Common range of values for the kernel matching propensity score.

**Figure 2.**
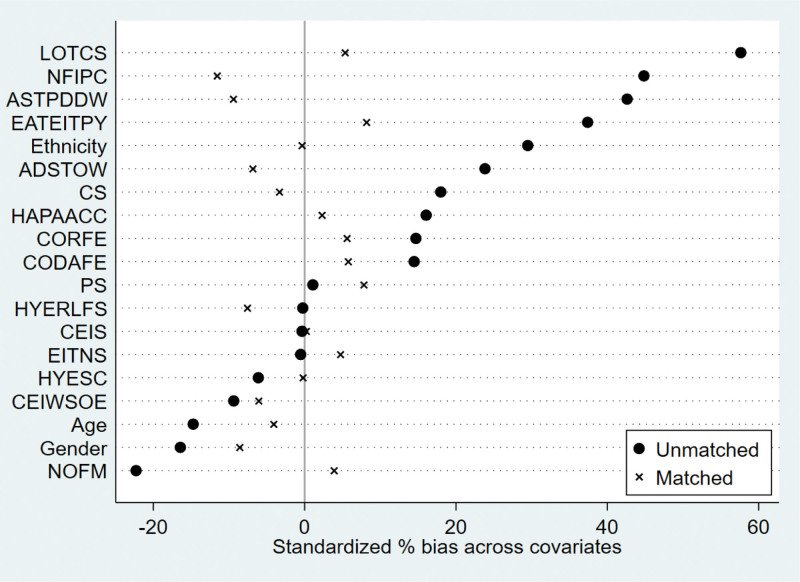
Plot of standardized deviations for each variable of kernel matching.

**Figure 3.**
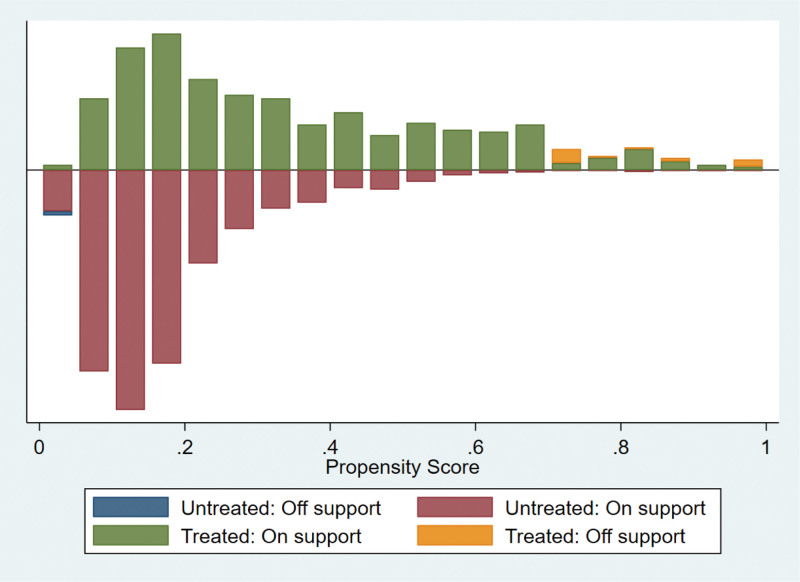
Common range of values for the radius matching propensity score.

**Figure 4.**
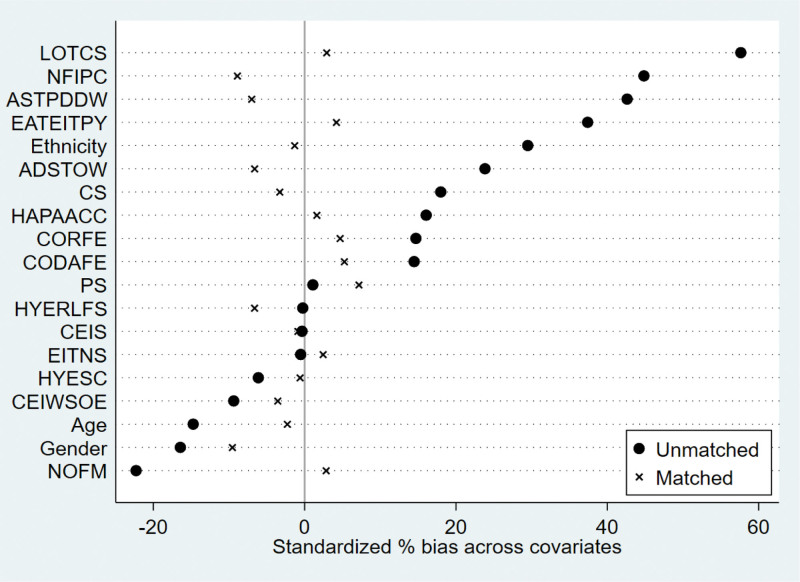
Plot of standardized deviations for each variable of radius matching.

**Figure 5.**
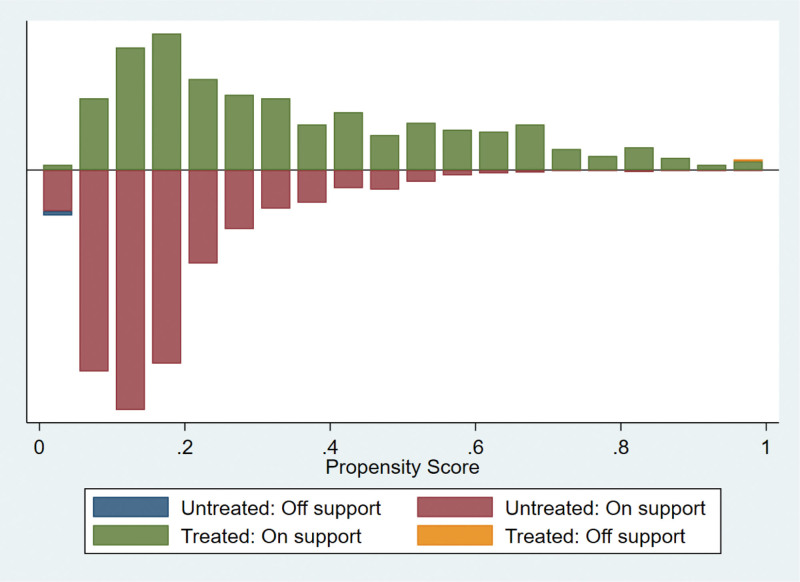
Common range of values for the neighbor matching (1:3) propensity score.

**Figure 6.**
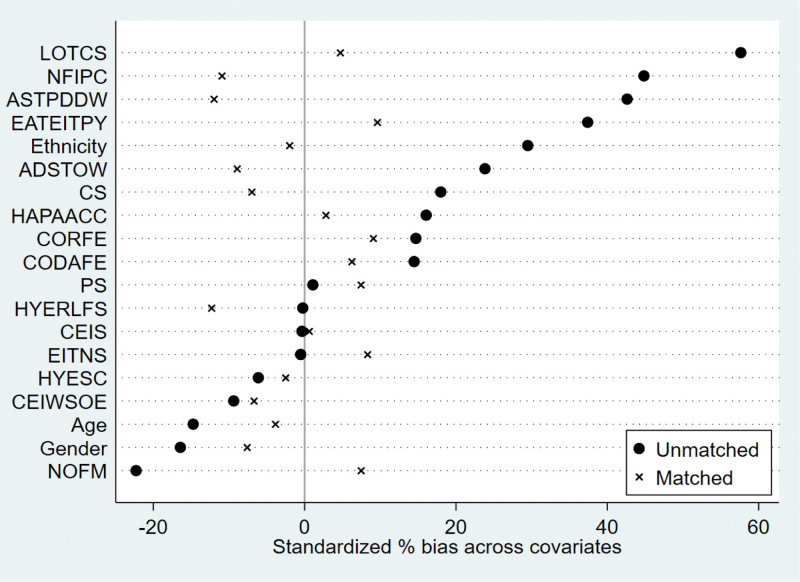
Plot of standardized deviations for each variable of neighbor matching (1:3).

We used calculated propensity scores to match 564 students who participated in extracurricular tutoring with 2003 who did not. To ensure the robustness of the results, we employed 3 common matching methods, including kernel matching, radius matching, and nearest neighbor matching. The kernel matching successfully matched 553 pairs of samples, the radius matched 538 pairs of samples, and the nearest neighbor matched 553 pairs of samples. The overall matching success rate was close to 100%, indicating that the 3 methods had a good overall matching effect. The standard deviation of the 2 samples after PSM should be <5%, and the independent samples t-test for each covariate between the control and treatment groups should no longer be significant. In addition, there were no significant differences in the 19 covariates between the control and treatment groups after matching (Table 4 and Figs. 1–6), indicating that these covariates did not provide any valid predictive information for participation in extracurricular tutoring after matching. Therefore, it passes the overall balance test.

**Table 4:** The *t* test of covariates between treatment and control group after matching.

Variables	Kernel matching		Radius matching		Neighbor matching (1:3)	
	*t* value	*P* value	*t* value	*P* value	*t* value	*P* value
Age (yr)	−0.680	.494	−0.380	.704	−0.650	.519
Gender	−1.420	.155	−1.570	.118	−1.260	.207
Ethnicity	−0.080	.940	−0.280	.779	−0.430	.665
Number of family members	0.680	.499	0.480	.628	1.340	.182
Net family income per capita (KCNY)	−1.330	.184	−1.110	.269	−1.350	.177
Education and training expenditure in the past year (KCNY)	1.170	.242	0.620	.532	1.380	.169
Change of residence for enrollment	0.860	.390	0.700	.483	1.430	.152
Change of domicile address for enrollment	0.830	.409	0.790	.430	0.900	.368
Currently enrolled in school	0.030	.973	−0.150	.882	0.100	.921
Currently enrolled in which stage of education	−1.010	.312	−0.590	.558	−1.110	.265
Locality of the current school	0.790	.431	0.420	.672	0.690	.488
Public school	1.240	.215	1.130	.258	1.180	.237
Enroll in the nearest school	0.780	.438	0.390	.696	1.350	.177
Class size	−0.560	.577	−0.540	.588	−1.160	.245
Hold a position as a class cadre	0.370	.710	0.260	.798	0.450	.650
Average daily study time on weekdays (h)	−1.040	.300	−1.000	.317	−1.340	.181
Average study time per day during weekends (h)	−1.280	.199	−0.960	.337	−1.610	.107
Have you ever requested leave from school	−1.220	.224	−1.060	.289	−1.940	.053
Have you ever skipped class	−0.040	.966	−0.110	.909	−0.450	.650

### 5.4. Sensitivity analysis

Rosenbaum proposed that the purpose of sensitivity analysis was to explore the extent to which hidden selection bias could alter the outcome of the treatment effect obtained,^[43,44]^ and he therefore suggested that sensitivity analysis must be performed. The higher the sensitivity value, the lower the sensitivity. In general, if the sensitivity value is greater than or equal to 2, the sensitivity of the study is low and the results are reliable. In this study a sensitivity analysis was carried out after matching using the Rbounds module in Stata and a sensitivity value of 2.7 was calculated. For this reason, we can assume that the results are reliable.

### 5.5. Final results

The ATT of an intervention can be estimated based on the results of the PSM.^[45–47]^ We estimated the ATT and standard errors using the results of the 3 matching methods. After PSM, adolescent students who participated in extracurricular tutoring (treatment group) had distinctly higher levels of cognitive ability than those who did not. In all 3 matching methods, there were apparent improvements in students’ word test levels by 1.221, 1.159, and 1.171, and math test levels by 0.767, 0.790, and 0.659 (Table 5).

**Table 5:** Average treatment effect on the treated in three matching methods

Matching method	Treated group		Control group		ATT		Standard error	
	Word test	Math test	Word test	Math test	Word test	Math test	Word test	Math test
Kernel matching	26.144	13.922	24.923	13.155	1.221***	0.767**	0.368	0.305
Radius matching	26.163	13.894	25.004	13.104	1.159***	0.790**	0.360	0.298
Neighbor matching (1:3)	26.145	13.922	24.974	13.263	1.171***	0.659*	0.386	0.328

Radius matching sets the matching radius to 1/4 standard error of the propensity value.

ATT, average treatment effect on the treated.

* *P* < .05,

** *P* < .01,

*** *P* < .001.

## 6. Discussion

This study aims to use Propensity Score Matching (PSM) analysis to investigate the evidence of the impact of extracurricular tutoring on the cognition of adolescents. First, this study examines the impact of adolescents participation in extracurricular tutoring on their cognition by using data from the 2018 China Family Panel Studies (CFPS). Participation of adolescents in extracurricular tutoring exhibited higher cognitive abilities. These findings confirm the significant influence of extracurricular tutoring on the cognitive development of adolescents.

This study provides more robust evidence to support the findings of many related studies on this topic. Tan et al^[48]^ reported that private tutoring can positively impact academic performance and reduce the workload of mainstream teachers, but can also widen the gap between students and increase pressure to engage in tutoring services. Conversely, Bray et al^[49]^ opposed supplementing school work with private tutoring due to it depriving students of their originality and initiative. Furthermore, previous studies have found negative correlations between the time spent participating in extracurricular activities, such as tutoring, interest classes, watching TV, surfing the Internet, playing games, and adolescent students cognitive ability.^[50]^

The above findings may be due to overestimating the effect of covariates. However, this study achieved an ideal control group by exact matching, meeting the basic requirements for causal inference between the treatment and control groups. As a result, this study provides robust evidence that extracurricular tutoring can improve the cognitive performance of adolescent students.

This study utilizes a novel approach to further understand and validate the impact of extracurricular tutoring on cognitive development from a methodological standpoint. Observational studies based on survey data lack randomization, resulting in selection bias and insufficient evidence for causal inference. To address this issue, this study employed PSM as a convenient and intuitive method, which is increasingly popular in different social science fields. An appropriate set of confounding covariates can be used for exact matching. This study identified 19 covariates from the literature that influence adolescent students cognitive abilities and decisions to participate in extracurricular tutoring. Additionally, no significant differences were found between the matched control and treatment groups, demonstrating successful matching and minimized selection bias.

Despite China’s high level of compulsory education coverage, true educational equity has yet to be achieved, and extracurricular tutoring exacerbates this inequity. The government can implement measures to address this, such as improving school hardware facilities, strengthening teacher training, and enriching teaching methods, providing students with equitable access to quality and homogeneous educational resources during school hours, promoting adolescent students cognitive development. Based on the above study’s results, this paper proposes 2 recommendations. First, the government should provide tutorial assistance to disadvantaged young students, promoting educational equity and healthy development. Second, the government should allocate education resources in a rational and effective manner, particularly by increasing investment in less developed areas, to achieve maximum educational equity.

## 7. Limitations

There were several limitations to this study that should be considered in interpreting the findings. First, while 19 covariates were used to control for potential confounding factors, it is possible that other important variables may play a role in adolescent students cognitive development and their participation in extracurricular tutoring. On the 1 hand, the use of secondary survey data may have limited the selection of variables included in the analysis. On the other hand, the diverse and complex factors that influence youth participation in extracurricular tutoring make it challenging to study them comprehensively. However, the 19 covariates selected were deemed satisfactory in covering most aspects affecting adolescent students participation and cognitive ability. Second, in addition to the PSM method, other statistical techniques can be used to address selection bias, such as regression adjustment and instrumental variables. Therefore, combining multiple methods for cross-validation will definitely improve the reliability and persuasiveness of the conclusions. Finally, it is important to note that the study utilized the most recent dataset available, which was the 2018 database, in order to provide current and relevant findings. However, this limitation also means that the study’s conclusions may not be generalizable to other time periods.

## 8. Conclusion

Our research results indicate that, after using PSM matching to control confounding variables, the cognitive ability level of teenagers who participated in extracurricular tutoring was significantly higher than those who did not participate in extracurricular tutoring. These findings suggest that the government should provide tutoring support to disadvantaged adolescent students as part of their efforts to promote healthy and equitable education development.

To further achieve equity in education, educational resources should be allocated rationally and effectively, with a particular focus on increasing investment in under-developed areas. Such measures will facilitate educational equity and ultimately better promote cognitive development among adolescent students.

## Acknowledgements

The authors thank the China Family Panel Studies (CFPS) team for their contribution to the collection of data. Additionally, we thank the participants for their consent to participate in the study.

## Author contributions

**Conceptualization:** Qi Zhang.

**Methodology:** Wenlong Wang.

**Project administration:** Zhihong Liu.

**Resources:** Jiafei Yang, Zhihong Liu.

**Software:** Wenlong Wang.

**Supervision:** Jiafei Yang.

**Writing – original draft:** Qi Zhang.

**Writing – review & editing:** Qi Zhang.

## References

[1] Zhang Y Ma X Wang L. The determinants of private tutoring participation for mathematics in China: focusing on the role of student metacognition. Front Psychol. 2020;11:603. 32328013 10.3389/fpsyg.2020.00603 PMC7153502

[2] Noh JW Kim J Cheon J. Relationships between extra-school tutoring time, somatic symptoms, and sleep duration of adolescent students: a panel analysis using data from the Korean Children and Youth Panel Survey. Int J Environ Res Public Health. 2020;17:8037. 33142769 10.3390/ijerph17218037 PMC7663676

[3] Byun SY Park H. The academic success of East Asian American Youth: the role of shadow education. Sociol Educ. 2012;85:40–60. 24163483 10.1177/0038040711417009 PMC3806291

[4] Briggs DC. The effect of admissions test preparation: evidence from NELS:88. Chance. 2012;14:10–8.

[5] Guill K Lüdtke O Köller O. Assessing the instructional quality of private tutoring and its effects on student outcomes: analyses from the German National Educational Panel Study. Br J Educ Psychol. 2020;90:282–300. 31004348 10.1111/bjep.12281 PMC7317363

[6] Bray M Kwok P. Demand for private supplementary tutoring: conceptual considerations, and socio-economic patterns in Hong Kong. Econ Educ Rev. 2003;22:611–20.

[7] Tansel A Bircan F. Demand for education in Turkey: a tobit analysis of private tutoring expenditures. Econ Educ Rev. 2006;25:303–13.

[8] Zhang Y. Does private tutoring improve students National College entrance exam performance? – A case study from Jinan, China. Econ Educ Rev. 2013;32:1–28.

[9] Wang X. Summary of survey on stress reduction policies and extracurricular tutoring for primary school students in Baoding City [in Chinese]. EconTrade. 2017:291.

[10] Gray R Gow AJ. How is musical activity associated with cognitive ability in later life? Neuropsychol Dev Cogn B Aging Neuropsychol Cogn. 2020;27:617–35. 31549569 10.1080/13825585.2019.1660300

[11] Villeneuve EF Hajovsky DB Mason BA. Cognitive ability and math computation developmental relations with math problem solving: An integrated, multigroup approach. Sch Psychol Q. 2019;34:96–108. 29985022 10.1037/spq0000267

[12] Sala G Tatlidil KS Gobet F. Video game training does not enhance cognitive ability: a comprehensive meta-analytic investigation. Psychol Bull. 2018;144:111–39. 29239631 10.1037/bul0000139

[13] Zhao J Wu M Zhou L. Cognitive psychology-based artificial intelligence review. Front Neurosci. 2022;16:1024316. 36278021 10.3389/fnins.2022.1024316 PMC9582153

[14] Yeh YH Myerson J Green L. Delay discounting, cognitive ability, and personality: what matters? Psychon Bull Rev. 2021;28:686–94. 33219456 10.3758/s13423-020-01777-w PMC8068578

[15] Bever TG. How cognition came into being. Cognition. 2021;213:104761. 34148649 10.1016/j.cognition.2021.104761

[16] Malanchini M Rimfeld K Allegrini AG. Cognitive ability and education: how behavioural genetic research has advanced our knowledge and understanding of their association. Neurosci Biobehav Rev. 2020;111:229–45. 31968216 10.1016/j.neubiorev.2020.01.016 PMC8048133

[17] Bottigliengo D Lorenzoni G Ocagli H. Propensity score analysis with partially observed baseline covariates: a practical comparison of methods for handling missing data. Int J Environ Res Public Health. 2021;18:6694. 34206234 10.3390/ijerph18136694 PMC8293809

[18] Yuan W Jiang M Gong S. How to improve the cognitive health of middle-aged and elderly people: evidence from China family panel studies. Front Public Health. 2022;10:799255. 35186840 10.3389/fpubh.2022.799255 PMC8855359

[19] Li Z Qin W Patel V. Associations of parental depression during adolescence with cognitive development in later life in China: a population-based cohort study. PLoS Med. 2021;18:e1003464. 33428637 10.1371/journal.pmed.1003464 PMC7799791

[20] Xu M Wang Y Yao S. One-year prevalence of perceived medical errors or near misses and its association with depressive symptoms among Chinese Medical Professionals: a propensity score matching analysis. Int J Environ Res Public Health. 2022;19:3286. 35328969 10.3390/ijerph19063286 PMC8949244

[21] Oichi T Oshima Y Chikuda H. In-hospital complication rate following microendos- copic versus open lumbar laminectomy: a propensity score-matched analysis. Spine J. 2018;18:1815–21. 29567515 10.1016/j.spinee.2018.03.010

[22] Benedetto U Head SJ Angelini GD. Statistical primer: propensity score matching and its alternatives. Eur J CardioThorac Surg. 2018;53:1112–7. 29684154 10.1093/ejcts/ezy167

[23] Pak SJ Kim YI Yoon YS. Short-term and long-term outcomes of laparoscopic vs open ileocolic resection in patients with Crohn’s disease: propensity-score matching analysis. World J Gastroenterol. 2021;27:7159–72. 34887635 10.3748/wjg.v27.i41.7159 PMC8613650

[24] Wang J. To use or not to use propensity score matching? Pharm Stat. 2021;20:15–24. 32776719 10.1002/pst.2051

[25] Wu Y Hu H Cai J. Association of hypertension and incident diabetes in Chinese adults: a retrospective cohort study using propensity-score matching. BMC Endocr Disord. 2021;21:87. 33926442 10.1186/s12902-021-00747-0 PMC8082672

[26] Austin PC. Optimal caliper widths for propensity-score matching when estimating differences in means and differences in proportions in observational studies. Pharm Stat. 2011;10:150–61. 20925139 10.1002/pst.433 PMC3120982

[27] Xu G Chen S. The current situation of extra-curricular tutoring for primary school students and its countermeasures: the case of Wenshan City, Yunnan Province [in Chinese]. J Wenshan Univ. 2016;29:117–20.

[28] Liu X. Does the academic performance benefit from the private supplementary tutoring?– Evidence from CEPS panel survey. Beijing Foreign Studies University [in Chinese]. 2021.

[29] Zhang Y Cui C He Y. Does private supplementary tutoring matter in Chinese students’ learning of mathematics: a longitudinal study. ZDM. 2022;54:737–47. 35313665 10.1007/s11858-022-01346-6 PMC8926893

[30] Guan Z. The effect of extra-curricular tuition on the academic performance of junior secondary school students-estimates based on the PSM-DID method [in Chinese]. China Econ Educ Rev. 2022;7:57–74.

[31] Niu C. The influence of extracurricular tutoring onthe gap of junior middle school students cognitive and non-cognitive abilities between urban and rural areas-an empirical research based on China education panel survey [in Chinese]. Central China Normal University. 2020;001366.

[32] Fraser MW Guo S. Propensity Score Analysis: Statistical Methods and Applications. Thousand Oaks: Sage; 2015.

[33] Zhang H Wang H Yan H. Impact of internet use on mental health among elderly individuals: a difference-in-differences study based on 2016-2018 CFPS Data. Int J Environ Res Public Health. 2021;19:101. 35010361 10.3390/ijerph19010101 PMC8749999

[34] Chen W Huang Y Riad A. Gender differences in depressive traits among rural and urban Chinese adolescent students: secondary data analysis of Nationwide survey CFPS. Int J Environ Res Public Health. 2021;18:9124. 34501714 10.3390/ijerph18179124 PMC8430502

[35] Tang S Yao L Ye C. Can health service equity alleviate the health expenditure poverty of Chinese patients? Evidence from the CFPS and China health statistics yearbook. BMC Health Serv Res. 2021;21:718. 34289849 10.1186/s12913-021-06675-y PMC8293547

[36] Chen Y Sun R. The impact of children’s gender on parent’s mental health and cognition – evidence from China. SSM Popul Health. 2022;18:101086. 35464614 10.1016/j.ssmph.2022.101086 PMC9019397

[37] Wu Y Hu H Cai J. A prediction nomogram for the 3-year risk of incident diabetes among Chinese adults. Sci Rep. 2020;10:21716. 33303841 10.1038/s41598-020-78716-1 PMC7729957

[38] Groenwold RHH Dekkers OM. Missing data: the impact of what is not there. Eur J Endocrinol. 2020;183:E7–9. 32688333 10.1530/EJE-20-0732

[39] Desai RJ Rothman KJ Bateman BT. A propensity-score-based fine stratification approach for confounding adjustment when exposure is infrequent. Epidemiology. 2017;28:249–57. 27922533 10.1097/EDE.0000000000000595 PMC5497217

[40] Pan CW Liu JH Wu RK. Disordered sleep and myopia among adolescents: a propensity score matching analysis. Ophthalmic Epidemiol. 2019;26:155–60. 30601071 10.1080/09286586.2018.1554159

[41] Liang J Hu Z Zhan C. Using propensity score matching to balance the baseline characteristics. J Thorac Oncol. 2021;16:e45–6. 34034891 10.1016/j.jtho.2020.11.030

[42] Austin PC Jembere N Chiu M. Propensity score matching and complex surveys. Stat Methods Med Res. 2018;27:1240–57. 27460539 10.1177/0962280216658920 PMC5843030

[43] Cheng Y. Overt and hidden bias in large observational studies. Am J Psychiatry. 2016;173:759–60. 27477134 10.1176/appi.ajp.2016.16050579

[44] Rosenbaum PR. Discussing hidden bias in observational studies. Ann Intern Med. 1991;115:901–5. 1952480 10.7326/0003-4819-115-11-901

[45] Ji X Cui N Liu J. Using propensity score matching with doses in observational studies: an example from a child physical abuse and sleep quality study. Res Nurs Health. 2019;42:436–45. 31674676 10.1002/nur.21991 PMC6858510

[46] Austin PC Cafri G. Variance estimation when using propensity-score matching with replacement with survival or time-to-event outcomes. Stat Med. 2020;39:1623–40. 32109319 10.1002/sim.8502 PMC7217182

[47] Austin PC Fine JP. Propensity-score matching with competing risks in survival analysis. Stat Med. 2019;38:751–77. 30347461 10.1002/sim.8008 PMC6900780

[48] Nilsson I. Confronting the shadow education system: what government policies for what private tutoring? Educ Rev. 2011;63:119–20.

[49] McVey M. Shadow education: private supplementary tutoring and its implications for policy makers in Asia. Int Rev Educ. 2012;58:809–11.

[50] Pan Y Zhou D Shek DTL. Participation in after-school extracurricular activities and cognitive ability among early adolescents in China: moderating effects of gender and family economic status. Front Pediatr. 2022;10:839473. 35372150 10.3389/fped.2022.839473 PMC8968855

